# The Expression of Key Guidance Genes at a Forebrain Axon Turning Point Is Maintained by Distinct Fgfr Isoforms but a Common Downstream Signal Transduction Mechanism

**DOI:** 10.1523/ENEURO.0086-19.2019

**Published:** 2019-04-09

**Authors:** Jung-Lynn Jonathan Yang, Gabriel E. Bertolesi, Stephanie Dueck, Carrie L. Hehr, Sarah McFarlane

**Affiliations:** Department of Cell Biology and Anatomy, Hotchkiss Brain Institute, Alberta Children’s Hospital Research Institute, Cumming School of Medicine, University of Calgary, Calgary, Alberta T2N 4N1, Canada

**Keywords:** axon guidance, fibroblast growth factor, MAPK, PI3K/AKT, retinal ganglion cell, signal transduction

## Abstract

During development the axons of neurons grow toward and locate their synaptic partners to form functional neural circuits. Axons do so by reading a map of guidance cues expressed by surrounding tissues. Guidance cues are expressed at a precise space and time, but how guidance cue expression is regulated, and in a coordinated manner, is poorly understood. Semaphorins (Semas) and Slits are families of molecular ligands that guide axons. We showed previously that fibroblast growth factor (Fgf) signaling maintains *sema3a* and *slit1* forebrain expression in *Xenopus laevis*, and these two repellents cooperate to guide retinal ganglion cell (RGC) axons away from the mid-diencephalon and on towards the optic tectum. Here, we investigate whether there are common features of the regulatory pathways that control the expression of these two guidance cues at this single axon guidance decision point. We isolated the *sema3a* proximal promoter and confirmed its responsiveness to Fgf signaling. Through misexpression of truncated Fgf receptors (Fgfrs), we found that *sema3a* forebrain expression is dependent on Fgfr2-4 but not Fgfr1. This is in contrast to *slit1*, whose expression we showed previously depends on Fgfr1 but not Fgfr2-4. Using pharmacological inhibitors and misexpression of constitutively active (CA) and dominant negative (DN) signaling intermediates, we find that while distinct Fgfrs regulate these two guidance genes, intracellular signaling downstream of Fgfrs appears to converge along the phosphoinositol 3-kinase (PI3K)-Akt signaling pathway. A common PI3K-Akt signaling pathway may allow for the coordinated expression of guidance cues that cooperate to direct axons at a guidance choice point.

## Significance Statement

Little is known about the mechanisms regulating axon guidance cue expression. Fibroblast growth factor (Fgf) signals maintain *semaphorin 3a* (*sema3a*) and *slit1* expression at a key guidance point for retinal ganglion cell (RGC) axons in the mid-diencephalon. We find that *sema3a* expression in the forebrain is promoted by a distinct set of Fgfrs, Fgfr2-4, from the Fgfr1 that maintains *slit1* levels. Interestingly, despite the differences in Fgfr dependency, the phosphoinositol 3-kinase (PI3K)-Akt signaling pathway is likely a shared downstream regulator of the expression of both guidance cues. These data argue that related but distinct receptors converge on a common signaling mechanism for coordinated control of a map of molecular cues that cooperate at a single guidance choice point to direct axon behavior.

## Introduction

Axon guidance, cell migration, and cell polarity are key processes required to integrate neurons into functional circuits (O’Donnell et al., 2009; Marín et al., 2010; Seiradake et al., 2016). To form connections, immature neurons respond to molecular cues that determine the position of their cell body and axonal and dendritic processes. These cues include the semaphorins (SEMAs), slits, netrins, and ephrins (Dickson, 2002; Seiradake et al., 2016). While the roles of these molecules are known, the mechanisms that regulate their expression are poorly understood.

The growth cones of axons read redundant molecular cues to navigate through the nervous system and change their direction at axon guidance choice points. For instance, multiple distinct molecules guide the crossing of dorsal commissural interneuron axons at the spinal cord midline, including several members of each of the main axon guidance families (de Ramon Francàs et al., 2017). An interesting question is whether unique or common regulatory pathways control the expression of guidance cues that cooperatively direct axonal trajectories at a single guidance choice point. Our previous work in the *Xenopus* forebrain provides a model to address this issue. We found that fibroblast growth factor (Fgf) signaling maintains the expression of two guidance cues, *sema3a* and *slit1*, which work together to repel retinal ganglion cell (RGC) axons in a caudal direction from an intermediate target within the diencephalon and on toward the optic tectum (Atkinson-Leadbeater et al., 2010); when either Fgf signaling, or both *sema3a* and *slit1* expression, are inhibited, many RGC axons fail to navigate away from this guidance choice point. We identified previously that signaling through Fgf receptor 1 (Fgfr1) maintains forebrain *slit1* expression (Yang et al., 2018). By determining through which Fgfr *sema3a* expression is regulated, and the downstream signaling pathway(s) that control the expression of both guidance cues, we set out to understand how a complete map of guidance cues is established that control the behavior of axons at a select axonal choice point.

FGFs regulate cell proliferation, migration, survival, and differentiation (Goetz and Mohammadi, 2013; Ornitz and Itoh, 2015), and through FGFRs activate well-known intracellular signaling cascades to change gene expression, including the mitogen-activated protein kinase (MAPK), phosphoinositol 3-kinase (PI3K)-AKT, and phospholipase Cγ (PLCγ) pathways (Musci et al., 1990; Klint and Claesson-Welsh, 1999; Wiedemann and Trueb, 2000). Here we identify an Fgf-responsive *sema3a* promoter and show that Fgfr2-4 and not Fgfr1 regulate *sema3a* expression. Interestingly, while distinct Fgfrs promote *sema3a* and *slit1* (Yang et al., 2018) forebrain expression, they likely employ a common PI3K-Akt signaling pathway. Thus, distinct extrinsic signaling inputs can converge on a common intracellular signal transduction pathway to coordinate the appropriate expression of redundant guidance cues that direct growth cone behavior at a guidance choice point.

## Materials and Methods

### Animals

*Xenopus laevis* oocytes, collected from adult females (Nasco) injected with human chorionic gonadotrophin (Chorulon, Intervet), were fertilized *in vitro*. Embryos developed at 16°C in 0.1× Marc’s modified Ringer’s solution (MMR; 0.1 M NaCl, 2 mM KCl, 1 mM MgCl_2_, and 5 mM HEPES, pH 7.5) and were staged according to Faber and Nieuwkoop (1994). Animal protocols were approved by the University of Calgary Animal Care Committee.

### Plasmid constructs

The *sema3a* upstream flanking nucleotides (–2930 to +63; GenBank accession number KP322598) were amplified by PCR from *X. tropicalis* hepatic genomic DNA using the forward primer 5’-GGTGCCTCATGGGTCAGATTG-3’ and reverse primer 5’-GTGTGTGCAAAGGCAGCAATG-3’. The first nucleotide transcribed in the *sema3a* mRNA was assigned the index +1. A PCR-amplified product using Master Mix (Thermo Fisher) was captured into the *pCRII-TOPO-TA* cloning vector (Life Technologies) according to the manufacturer’s instructions. *sema3a* deletion fragments were isolated by restriction enzymes and ligated upstream of firefly luciferase cDNA in the *pGL3* basic (Promega) vector. Primer synthesis and DNA sequencing were conducted at the University of Calgary DNA Core Facility. The *Xenopus* expression constructs to inhibit Fgfrs were *pCS2-DNfgfr1* (Ueno et al., 1992), *pCS108-DNfgfr2*, *pCS108-sfgfr3*, and *pCS2MTC-DNfgfr4* (Golub et al., 2000; Atkinson-Leadbeater et al., 2010, 2014). Other constructs used were *pCS108-BRAFV600E* (courtesy of Carol Schuurmans, University of Toronto; Boehm et al., 2007; Tachibana et al., 2016), *pCS2-MEKK1+* and *pCS2-MEKK1-KM* (provided by Ángel Nebreda, IRB Barcelona; Ben Messaoud et al., 2015), *^T308D/S473D^PKB* and *^T308A/S473A^PKB* (courtesy of Florian Lang, Universität Tübingen; Palmada et al., 2005), *AKT-myr* (provided by Jing Yang, University of Illinois; Jin et al., 2015), and *pEYFP-PLCγ* and *phRluc-N1-FGFR1Y766F* (provided by Anne-Françoise Burnol, Institut Cochin; Browaeys-Poly et al., 2010).

### Cell culture and luciferase assay

XTC cells (Pudney et al., 1973; RRID: CVCL_5610), an *X. laevis* fibroblast cell line provided by Manfred Lohka, University of Calgary, were maintained in 60% Leibovitz’s L-15 Medium (Gibco) supplemented with 10% fetal bovine serum (FBS). Cells were seeded into 96-well plates (Greiner) 24 h before transfection. Transfections were performed with Lipofectamine 2000 (Life Technologies) in medium without FBS, according with the manufacturer’s specifications; each well was co-transfected with 100-ng firefly luciferase (*luc*) reporter plasmid, 75-ng *Renilla* plasmid (transfection control, in which the ubiquitous HSV-thymidine kinase promoter drives *Renilla* luciferase expression), and, where required, 50 ng of an expression plasmid or *pCS2-GFP* (as control). The medium was replaced with medium containing FBS 6 h after transfection. Where indicated, 3-[3-(2-carboxyethyl)-4-methylpyrrol-2-methylidenyl]-2-indolinone (SU5402; Sigma) was added to wells. Cells were harvested for luciferase assay 48 h after transfection. Luciferase activity was quantified by the Dual-Glo Luciferase Assay System (Promega) as per the manufacturer’s instructions on a FilterMax F5 Multi-Mode Microplate Reader (Molecular Devices). To quantify promoter induction as relative light units, firefly luciferase activity was first normalized against *Renilla* activity to control for transfection efficiency, and then normalized against the promoterless *pGL3* basic vector.

### *Xenopus* brain electroporation

Brain electroporation of expression plasmids was performed on stage 27/28 embryos anaesthetized in 0.4-mg/ml tricaine methanesulfonate (Sigma-Aldrich) in 1× modified Barth’s saline (MBS; 0.7 mM CaCl_2_, 5 mM HEPES, 1 mM KCl, 1 mM MgSO_4_, 88 mM NaCl, and 2.5 mM NaHCO_3_; pH 7.8; Haas et al., 2002; Chen et al., 2007). Briefly, a 1-μg/μl plasmid DNA solution was microinjected into the central forebrain ventricle using a Picospritzer II (General Valve). Platinum electrodes, spaced 3 mm apart and connected to a Grass Technologies S44 system, were placed on either side of the embryo’s head to deliver 10 50-ms 35-V pulses. Electroporated embryos were reared in 0.1× MMR for development to stage 32.

### *In situ* hybridization (ISH)

Digoxigenin (DIG)-labeled and fluorescein-labeled riboprobes were prepared and used for ISH as described previously (Atkinson-Leadbeater et al., 2010; Yang et al., 2018). Briefly, riboprobes were transcribed *in vitro* using SP6 or T7 polymerase (Roche), DIG-labeled or fluorescein-labeled ribonucleotides (Roche), and linearized plasmid templates *pBSK-xfgfr1*, *pBSK-xBek-ec*, *pBSK-xfgfr3*, *pBSK-xfgfr4*, *pCRII-xsema3A*, and *pCMV-SPORT6-slit1* (Golub et al., 2000; Atkinson-Leadbeater et al., 2009, 2010, 2014). The specificity of all riboprobes was assessed through sense controls. For color development of wholemount ISH, tissues were incubated with anti-DIG alkaline phosphatase-conjugated Fab fragments (Roche catalog #11 093 274 910; RRID: AB_2313640) and stained with BM Purple (Roche; Sive et al., 2000). For double fluorescent ISH (dFISH) on sectioned tissue, samples were incubated with anti-DIG peroxidase-conjugated (Roche catalog #11 207 733 910; RRID: AB_514500) or anti-fluorescein peroxidase-conjugated (Roche catalog #11 426 346 910; RRID AB_840257) Fab fragments and stained with the TSA Plus Fluorescein Evaluation kit (PerkinElmer) and the TSA Cyanine 3 System (PerkinElmer).

### Immunohistochemistry

Embryos were fixed in 4% paraformaldehyde, cryoprotected in 30% sucrose solution, and mounted in OCT compound (Tissue Tek, Sakura Finetek, Inc.) for cryosectioning to 12-µm thickness. The tissues were permeabilized in 0.5% Triton X-100 (Sigma-Aldrich) and blocked in 5% goat serum (Thermo Fisher) in PBS for 30 min at room temperature. Primary antibodies (1:500 dilution) were against hemagglutinin (HA; Covance catalog #MMS-101P; RRID: AB_2565006), green fluorescent protein (GFP; Invitrogen catalog #A11120; RRID: AB_221568), and myc (Santa Cruz Biotechnology catalog #sc-789; RRID: AB_631274). The secondary antibodies conjugated to Alexa Fluor 488 (1:1000 dilution) were goat anti-mouse (Abcam catalog #ab150113; RRID: AB_2576208) and goat anti-rabbit (Abcam catalog #ab150077; RRID: AB_2630356). Nuclei were stained with 4,6-diamidino-2-phenylindole (DAPI; 1:1000; Life Technologies).

### *In vivo*-exposed brain preparation

The skin and dura overlying the left side of the brain of stage 32 or stage 33/34 embryos were removed to expose the entire anterior brain to as far as the posterior optic tectum (Chien et al., 1993). Embryos were incubated in specific inhibitors against Fgfrs, Mek, PI3K, and PLCγ in 1× MBS at room temperature for 5 h: SU5402, 1,4-diamino-2,3-dicyano-1,4-bis(*o*-aminophenylmercapto) butadiene monoethanolate (U0126; Calbiochem), 2-(4-morpholinyl)-8-phenyl-4H-1-benzopyran-4-one (LY294002; Calbiochem), and 1-[6-[((17β)-3-methoxyestra-1,3,5[10]-trien-17-yl)amino]hexyl]-1H-pyrrole-2,5-dione (U73122; Calbiochem), respectively. The control incubation was 1× MBS supplemented with 0.2% dimethyl sulfoxide (DMSO). For ISH, embryos were fixed in 4% paraformaldehyde. For RT-qPCR, the anterior brains were collected into TRIzol (Life Technologies). For optic tract tracing, the RGC axons were anterogradely labeled with horseradish peroxidase (HRP) at stage 40 (Atkinson-Leadbeater et al., 2010). To quantify the axon stall phenotype, the width of the optic tract post mid-diencephalic turn was represented as a ratio to the width of the optic tract at the mid-diencephalic turn.

### Reverse transcription (RT)-PCR

Total RNA was extracted from *X. laevis* forebrains and XTC cells using TRIzol and chloroform and purified with the GeneJET RNA Purification kit (Thermo Fisher). RNA quality was assessed on the Agilent 2200 TapeStation (University of Calgary Center for Health Genomics and Informatics). The cDNA template was prepared from 100-ng total RNA in 20-µl reactions using SuperScript II Reverse Transcriptase (Life Technologies) and primed with oligodT (Life Technologies). The composition of quantitative RT-PCR (RT-qPCR) reactions was 3-µl cDNA solution, 1× QuantiTect SYBR Green PCR kit (QIAGEN), 417 nM each of forward and reverse primers, and water up to 20 µl. RT-PCR primers for *fgfr1-4* and RT-qPCR primers (Table 1) for *slit1* and *spry1*, and the reference genes *β*-actin (*actb*), tubulin *β* class I (*tubb*), and light chain dynein (*dynll1*), were validated previously (Brunsdon, 2015; Yang et al., 2018). We also validated RT-qPCR primers for *sema3a* and *slit2* for high efficiency and specificity (data not shown). RT-qPCR reactions were run on the CFX Connect Real-Time PCR Detection System (Bio-Rad) and the CFX Manager Software v3.1 (Bio-Rad). Each RT-PCR and RT-qPCR reaction was thermocycled as initial denature (95°C, 10 min), denature (95°C, 15 s), anneal (54°C, 35 s), and elongation (72°C, 30 s) for a total of 40 cycles. The RT-qPCR product was heated to 95°C in 0.5°C increments for melt curve analysis.

**Table 1.** RT-qPCR primers and their properties for quantifying gene expression

Gene	Primers	Amplicon	Efficiency	Amplicon *T*_melt_ (°C)
actb*	Forward, GTTGATAATGGATCTGGTATGTGC	Nucleotides 89–201, GenBank accession number NM_001088953.1	0.9401	83.0
	Reverse, ATTCCAACCATGACACCCTGA			
dynll1◊	Forward, TGCTACTCAGGCACTGGAGA	Nucleotides 127–239, GenBank accession number NM_001171695.1	0.9576	77.5
	Reverse, AATTCCTTCCCACAATGCAA			
sema3a	Forward, TGAAGAACGGGGAAGACTTTATG	Nucleotides 189–288, GenBank accession number NM_001085855.1	0.9738	78.5
	Reverse, TGTTACAGGCCACAATATCTTTTG			
slit1*	Forward, TGCTGAGCGTAAACTTTGTGG	Nucleotides 3756–3855, GenBank accession number NM_001087109.1	0.9931	81.0
	Reverse, TCCGCTGTTGACACCTGAAG			
slit2	Forward, CCAGGAGCATTCTCACCATACA	Nucleotides 1510–1628, GenBank accession number NM_001087668.1	0.9220	80.0
	Reverse, TAGAGAACAAGGGAGTTGAGTGA			
spry1 a/b*	Forward, CAACATGGCATTGGTGGTTCAT	Nucleotides 16–110, GenBank accession number NM_001137585.1; nucleotides 72–166, GenBank accession number NM_001159681.1	0.9490	82.0
	Reverse, TTTGATCTGATCCAAGGACAAGATAG			
tubb*	Forward, GACCCTTTGGACAGATTTTCAGG	Nucleotides 300–405, GenBank accession number NM_001087257.1	0.9635	81.5

*Yang et al. (2018).

◊Brunsdon (2015).

### Western blotting

The brains of pharmacologically treated embryos and XTC cells were collected in 100 µL homogenization buffer [10% glycerol, 137 mM NaCl, 1.5 mM Na_3_VO_4_, 1% NP40, 0.1% sodium dodecyl sulfate (SDS), 20 mM Tris; pH 8.0, and Roche protease inhibitor mix diluted 1:100]; 10 µl of the homogenate was added to 200 µl of protein assay dye (Bio-Rad) diluted 1:4 and measured by the FilterMax F5 Multi-Mode Microplate Reader. Sample buffer (2% mercaptoethanol, 20% glycerol, and 4% SDS) was added to 5-µg protein samples loaded onto a 10% polyacrylamide gel. Samples were transferred to a PVDF membrane (Bio-Rad) and incubated in blocking solution containing primary antibodies (1:1000 dilution); rabbit anti-pERK (Cell Signaling Technology catalog #4370; RRID: AB_2315112), anti-pAKT (Cell Signaling Technology catalog #4060; RRID: AB_2315049), anti-GAPDH (Cell Signaling Technology catalog #5174; RRID: AB_10622025), anti-SEMA3A (Abcam catalog #ab23393; RRID: AB_447408), and goat anti-actin antibodies (Abcam catalog #ab8229; RRID: AB_306374) at room temperature for 1 h. ECL solution (GE Healthcare Bioscience) was used according to the manufacturer’s instructions after incubation with the secondary antibodies (1:500 dilution) HRP-conjugated goat anti-rabbit (Jackson ImmunoResearch catalog #111-035-144; RRID: AB_2307391) or donkey anti-goat (Jackson ImmunoResearch catalog #705-035-147; RRID: AB_2313587).

### Image processing and analysis

Microscope images were captured on the AxioCam HRc software (Carl Zeiss) on the Stemi SV II stereomicroscope (Carl Zeiss) and processed minimally in Photoshop (Adobe, 2017.0.0 release) for brightness and contrast. To evaluate co-expression of *fgfr* and *slit1* mRNAs with *sema3a* mRNA, ImageJ (1.51r; Schneider et al., 2012) was used to analyze the extent of overlap in the area of the gene expression domains in images of dFISH brain sections. The area of the *fgfr* or *slit1* domain is reported as a fraction of the area of the *sema3a* domain.

### Statistical analysis

For all quantitative datasets, the bars represent the mean with error bars representing the SEM from a total of *n* replicates in *N* independent experiments. After considering normality, datasets involving a control group and a treatment group were analyzed on GraphPad Prism 7 (RRID: SRC_002798) by using unpaired, two-tailed Student’s *t* test. Datasets involving a control group and multiple treatment groups were analyzed on GraphPad Prism 7 using one-way ANOVA with Dunnett’s or Bonferroni’s *post hoc* test. RT-qPCR data were analyzed on the Relative Expression Software Tool (REST) 2009 V2.0.13 (QIAGEN). The expression of *spry1*, *sema3a*, and *slit1/2* were normalized against the stably expressed reference genes *actb*, *tubb*, *dynll1*. For all statistical analyses, the threshold of significance was set at 0.05. The details of the statistical analyses are shown in Table 2.

**Table 2.** Statistical table

	Data structure	Type of test	95% confidence interval
a	Normal distribution	One-way ANOVA, Bonferroni's multiple comparison *post hoc* test	[–20.96, –1.920]
b	Normal distribution	Student’s *t*, two-tailed	[–1.786, 66.14]
c	Normal distribution	Student’s *t*, two-tailed	[–69.34, –7.449]
d	Normal distribution	One-way ANOVA, Dunnett’s multiple comparison *post hoc* test	[–23.38, 32.66]
e	Normal distribution	One-way ANOVA, Dunnett’s multiple comparison *post hoc* test	[18.43, 80.26]
f	Normal distribution	One-way ANOVA, Dunnett’s multiple comparison *post hoc* test	[18.77, 77.36]
g	Normal distribution (central limit theorem)	Randomization test, 2000 iterations	[0.155, 3.345]
h	Normal distribution (central limit theorem)	Randomization test, 2000 iterations	[0.048, 1.645]
i	Normal distribution (central limit theorem)	Randomization test, 2000 iterations	[0.077, 1.438]
j	Normal distribution (central limit theorem)	Randomization test, 2000 iterations	[0.062, 1.240]
k	Normal distribution (central limit theorem)	Randomization test, 2000 iterations	[0.049, 13.756]
l	Normal distribution (central limit theorem)	Randomization test, 2000 iterations	[0.056, 9.819]
m	Normal distribution (central limit theorem)	Randomization test, 2000 iterations	[0.022, 1.931]
n	Normal distribution (central limit theorem)	Randomization test, 2000 iterations	[0.019, 1.771]
o	Normal distribution (central limit theorem)	Randomization test, 2000 iterations	[0.033, 1.649]
p	Normal distribution (central limit theorem)	Randomization test, 2000 iterations	[0.367, 1.650]
q	Normal distribution (central limit theorem)	Randomization test, 2000 iterations	[0.011, 0.922]
r	Normal distribution (central limit theorem)	Randomization test, 2000 iterations	[0.024, 1.198]
s	Normal distribution (central limit theorem)	Randomization test, 2000 iterations	[0.361, 1.499]
t	Normal distribution (central limit theorem)	Randomization test, 2000 iterations	[0.016, 22.586]
u	Normal distribution (central limit theorem)	Randomization test, 2000 iterations	[0.022, 1.931]
v	Normal distribution (central limit theorem)	Randomization test, 2000 iterations	[0.024, 1.564]
w	Normal distribution (central limit theorem)	Randomization test, 2000 iterations	[0.662, 1.628]
x	Normal distribution (central limit theorem)	Randomization test, 2000 iterations	[0.473, 2.347]
y	Normal distribution (central limit theorem)	Randomization test, 2000 iterations	[0.676, 9.860]
z	Normal distribution (central limit theorem)	Randomization test, 2000 iterations	[0.595, 2.040]
aa	Normal distribution (central limit theorem)	Randomization test, 2000 iterations	[0.570, 2.160]
ab	Normal distribution (central limit theorem)	Randomization test, 2000 iterations	[0.461, 1.626]
ac	Normal distribution (central limit theorem)	Randomization test, 2000 iterations	[0.360, 1.574]
ad	Normal distribution (central limit theorem)	Randomization test, 2000 iterations	[0.432, 1.370]
ae	Normal distribution (central limit theorem)	Randomization test, 2000 iterations	[0.315, 1.722]
af	Normal distribution (central limit theorem)	Randomization test, 2000 iterations	[0.450, 1.469]
ag	Normal distribution (central limit theorem)	Randomization test, 2000 iterations	[0.281, 1.182]
ah	Normal distribution (central limit theorem)	Randomization test, 2000 iterations	[0.283, 1.425]
ai	Normal distribution (central limit theorem)	Randomization test, 2000 iterations	[0.469, 1.389]
aj	Normal distribution (central limit theorem)	Randomization test, 2000 iterations	[0.652, 3.255]
ak	Normal distribution (central limit theorem)	Randomization test, 2000 iterations	[0.480, 2.787]
al	Normal distribution (central limit theorem)	Randomization test, 2000 iterations	[0.856, 4.190]
am	Normal distribution (central limit theorem)	Randomization test, 2000 iterations	[0.812, 1.928]
an	Normal distribution (central limit theorem)	Randomization test, 2000 iterations	[0.530, 30.724]
ao	Normal distribution (central limit theorem)	Randomization test, 2000 iterations	[0.294, 1.836]
ap	Normal distribution	Student’s *t*, two-tailed	[0.480, 0.694]
aq	Normal distribution	Student’s *t*, two-tailed	[0.346, 0.617]

## Results

### *fgfr1-4* and *sema3a* co-express in the embryonic forebrain

**Figure 1.**
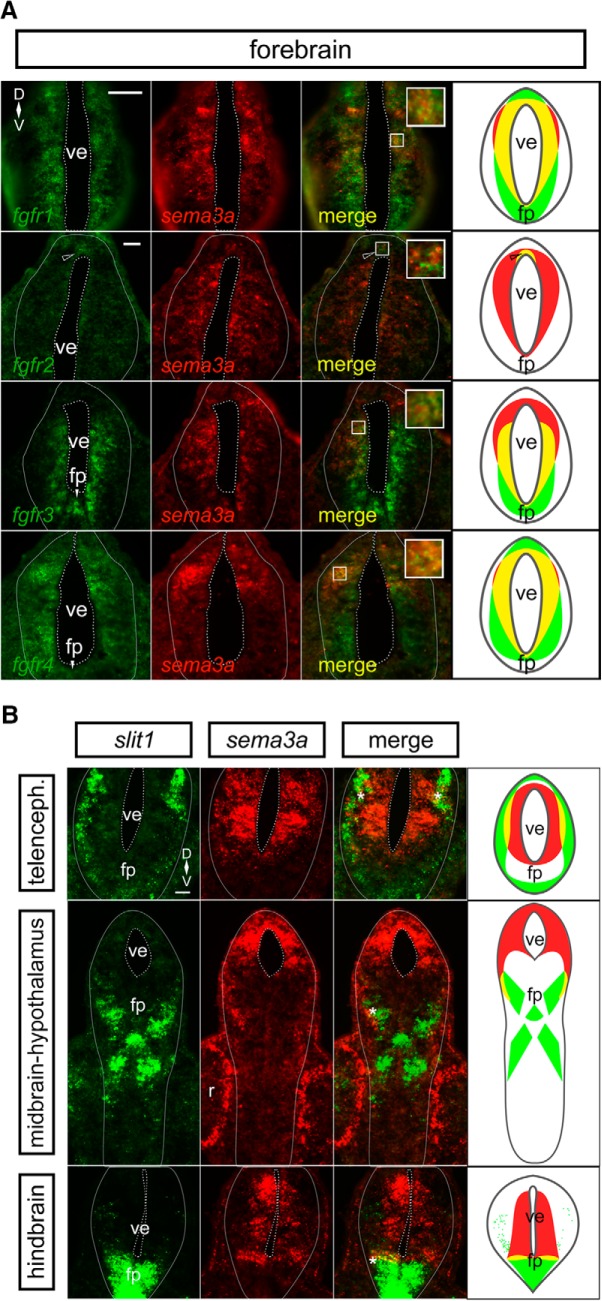
*sema3a* is co-expressed with *fgfrs* in the embryonic forebrain, but in complementary domains to *slit1*. ***A***, dFISH on transverse sections through the forebrain using specific antisense riboprobes against *fgfr1-4* (green) and *sema3a* (red). There is co-expression (yellow) of *sema3a* with all *fgfrs* in the ventricular zone of the forebrain. The insets reveal co-localization of dFISH signal. The rightmost column shows cartoons of the *fgfr* (green) and *sema3a* (red) domains, with co-expression in yellow. The *fgfr2* region of co-expression is restricted to the dorsal ventricular zone (unfilled arrowhead). ***B***, dFISH on transverse sections through the forebrain by using specific antisense riboprobes against *slit1* (green) and *sema3a* (red). *sema3a* is expressed by cells around the forebrain ventricle, whereas *slit1* is localized to the pial cells and the floor plate of the neural tube. There is some limited co-expression (yellow) of *sema3a* and *slit1* at the interface (asterisk) of the two expression domains. The rightmost column shows cartoons of the *slit1* (green) and *sema3a* (red) domains, with co-expression in yellow. Scale bars, 50 µm. fp, floor plate; ve, ventricle.

Sema3a and Slit1 cooperate as repellents to guide RGC axons past the mid-diencephalon and toward the midbrain target, the optic tectum. We found previously that Fgf signaling in the *Xenopus* forebrain maintains the expression of both guidance genes (Atkinson-Leadbeater et al., 2010), with Fgfr1 critical for regulating *slit1* expression (Yang et al., 2018). To investigate whether Fgfr1 also regulates forebrain *sema3a*, we first asked which *fgfr* isoforms are present alongside *sema3a* domains. We performed dFISH on transverse sections through the *X. laevis* forebrain (Fig. 1*A*) to observe *fgfr*-*sema3a* co-expression at stage 32, when RGC axons are growing toward the *sema3a* and *slit1* forebrain domains (Atkinson-Leadbeater et al., 2010). Of note, due to the distinct spatial expression patterns of *fgfrs* in the forebrain, we show representative sections where maximal co-expression occurred along the rostrocaudal axis of *sema3a* with the particular *fgfr* isoform. Unlike *slit1*, whose expression in the forebrain overlapped significantly only with that of *fgfr1* (Yang et al., 2018), the dFISH signal for all four *fgfr* isoforms coincided with some portion of the *sema3a* expression domain. For instance, both *fgfr1* and *sema3a* were expressed by the cells surrounding the ventricle of the forebrain, and *fgfr2* was expressed alongside *sema3a* at the apical (ventricular) face of the roof plate. In analysis of forebrain sections, the *fgfr1/2* signals covered 85% and 2%, respectively, of the *sema3a* domain. *sema3a* expression overlapped with both *fgfr3/4* in cells surrounding the ventricle. Overlap was not complete, in that the *fgfr3* region extended more ventrally to coincide with 57% of the *sema3a* domain, and that of *fgfr4* covered 90% of the *sema3a* domain within the dorsal neural tube. Altogether, the co-expression of *sema3a* with *fgfrs* supports a model whereby Fgfr signaling regulates cell-autonomously *sema3a* expression in the forebrain.

Since Sema3a and Slit1 cooperate to guide RGC axons (Atkinson-Leadbeater et al., 2010), we compared the spatial aspects of *sema3a* and *slit1* expression. By dFISH, we visualized the expression patterns of both genes in transverse sections along the rostrocaudal axis of the brain. Interestingly, given their cooperative role in RGC axon guidance, the *sema3a* and *slit1* domains were generally non-overlapping; *sema3a* expression was localized toward the ventricular face of the neural tube, while *slit1* mRNA was at the pial surface and in the floor plate (Fig. 1*B*). Of note, there was slight *sema3a*-*slit1* co-expression in cells at the interface of the expression domains of the two guidance genes, seen in the telencephalon, midbrain, and hindbrain coinciding with 17%, 8%, and 17%, respectively, of the *sema3a* domain. The distinct *fgfr*-*sema3a* and *fgfr*-*slit1* (Yang et al., 2018) co-localization patterns together with the non-overlapping *sema3a*-*slit1* expression in the forebrain suggest that unique regulatory mechanisms exist to control these two guidance cues.

### Fgf signaling induces *sema3a* promoter activity

**Figure 2.**
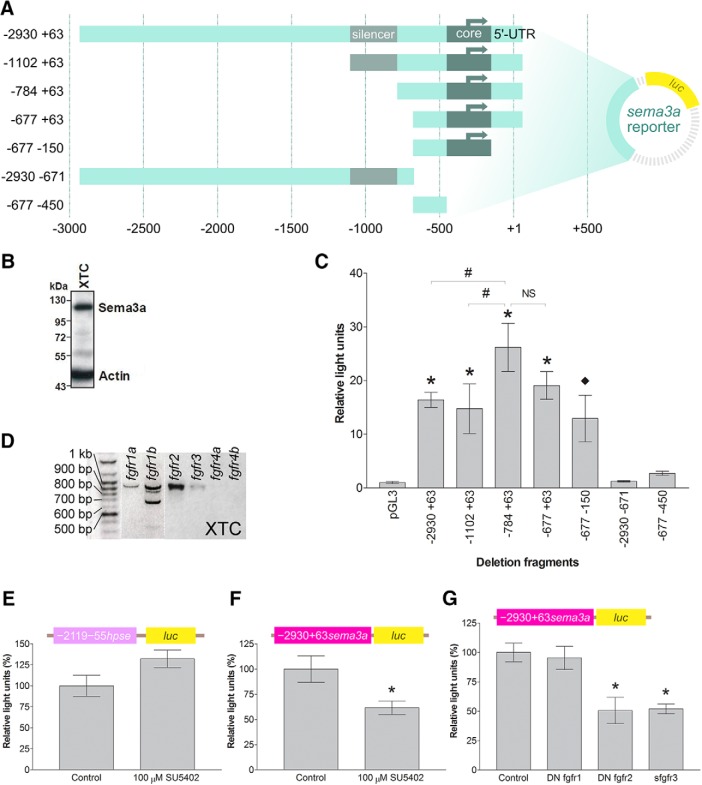
Characterization of the *sema3a* promoter. ***A***, Schematic of the *sema3a* deletion fragments inserted upstream of the firefly luciferase (*luc*) gene in the *pGL3* vector. ***B***, XTC cells express endogenous Sema3a protein by Western blotting. ***C***, The reporter constructs of the *sema3a* promoter fragments and the *Renilla* luciferase construct were co-transfected into XTC cells. Luminescence corresponding to each deletion fragment was normalized to *Renilla* to account for transfection efficiency and expressed as relative light units compared to the promoterless *pGL3* basic vector. Each bar represents mean ± SEM from *n* = 9 wells, *N* = 3. Statistical significance was determined by one-way ANOVA (α = 0.05) and the following *post hoc* tests: ^♦^*p* < 0.05 and *p < 0.01 by Dunnett’s multiple comparison test versus the control (*pGL3* vector) and #*p* < 0.05 by Bonferroni’s multiple comparison test between selected deletion fragments. ***D***, Expression of *fgfr* genes in XTC cells, shown by RT-PCR. XTC cells express all the *fgfr* genes (as verified by sequencing of amplicons) except for *fgfr4a/b*. Of note, the *fgfr1b* amplicons smaller than 900 bp may be alternatively spliced variants (Friesel and Dawid, 1991). ***E***, ***F***, The heparanase promoter construct (***E***, *n* = 12 wells, *N* = 2 for both bars), –2119 –55 *hpse::luc*, and the –2930 +63 *sema3a::luc* construct (***F***, *n* = 12 wells, *N* = 3 for both bars) were co-transfected into XTC cells in 96-well plates with the *Renilla* luciferase plasmid and treated with 100 µM SU5402. Bars reflect mean ± SEM; *p < 0.05, compared to DMSO control, two-tailed Student’s *t* test. ***G***, –2930 +63 *sema3a::luc* was co-transfected with truncated *fgfrs* in XTC cells (*N* = 7 for all bars; control *n* = 30 wells, DN *fgfr1 n* = 16 wells, DN *fgfr2 n* = 12 wells, *sfgfr3 n* = 14 wells). Bars reflect mean ± SEM; *p < 0.05 by ANOVA and Bonferroni *post hoc* test compared to *pCS2*-*GFP* transfection as control.

To examine the mechanisms of *sema3a* transcriptional regulation, we isolated the *sema3a* 5’-flanking sequence, and performed a deletion analysis to characterize the proximal promoter elements. Deletion fragments from the 5’- and 3’-end of the −2930 +63 *sema3a* sequence were inserted upstream of the luciferase (*luc*) reporter gene (Fig. 2*A*). These deletion constructs were co-transfected alongside *Renilla*, to normalize transfection efficiency, into XTC cells, an *X. laevis* fibroblast cell line. The XTC line was found to express Sema3a by Western blotting (Fig. 2*B*), suggesting *sema3a* regulatory mechanisms are in place. In the identified *sema3a* promoter there appeared to be silencing activity within −1102 to −784, as exclusion of these nucleotides increased reporter induction (*n* = 9 and *N* = 3 for all bars, compare constructs −1102 +63 and −784 +63, *p* < 0.05; Fig. 2*C*; row a, Table 2). The shortest deletion fragment with promoter activity was −677 −150. Since the −677 −450 fragment did not possess promoter activity, the *sema3a* core promoter was therefore located within nucleotides −450 to −150.

Next, we examined which *fgfr* isoforms could regulate the activity of the *sema3a* promoter. To confirm that the upstream regulatory sequence contained the necessary elements for responsiveness to Fgf signaling, we explored whether Fgfr signaling acted on the identified −2930 +63 *sema3a* sequence in XTC cells. Of note, by RT-PCR we found that XTC cells express *fgfr1-3*, but not *fgfr4* (Fig. 2*D*). Pharmacological inhibition of Fgfrs with 100 µM of Fgfr inhibitor SU5402 did not affect the activity of a heparanase promoter construct (Bertolesi et al., 2011; *n* = 12 and *N* = 2 for both bars, *p* = 0.062; Fig. 2*E*; row b, Table 2), but repressed significantly the activity of the −2930 +63 *sema3a::luc* reporter (*n* = 12 and *N* = 3 for both bars, *p* = 0.018; Fig. 2*F*; row c, Table 2). To identify the contribution of each Fgfr to *sema3a* transactivation, expression vectors for truncated Fgfrs (Ueno et al., 1992; Golub et al., 2000; Atkinson-Leadbeater et al., 2010, 2014) were co-transfected into XTC cells along with −2930 +63 *sema3a::luc* and *Renilla*. The dominant negative (DN) Fgfrs form non-functional heterodimers with wild-type Fgfrs, thereby eliminating signaling from their wild-type counterparts (Ueno et al., 1992), while soluble Fgfr3 (sFgfr3) sequesters specific Fgfs from binding endogenous Fgfrs (Fukuchi-Shimogori and Grove, 2001). Blocking Fgfr1 did not affect significantly the *sema3a* promoter activity (DN *fgfr1 n* = 16 wells and *N* = 7 vs control *n* = 30 wells and *N* = 7, *p* > 0.05; Fig. 2*G*; row d, Table 2), while DN *fgfr2* and *sfgfr3* lowered *sema3a* promoter activity to 50.7% (*n* = 12 wells and *N* = 7, *p* < 0.01; Fig. 2*G*; row e, Table 2) and 51.9% (*n* = 14 wells and *N* = 7, *p* < 0.01; Fig. 2*G*; row f, Table 2) of basal activity, respectively. Therefore, in XTC cells, the *sema3a* promoter is regulated in an Fgfr isoform-dependent fashion.

**Figure 3.**
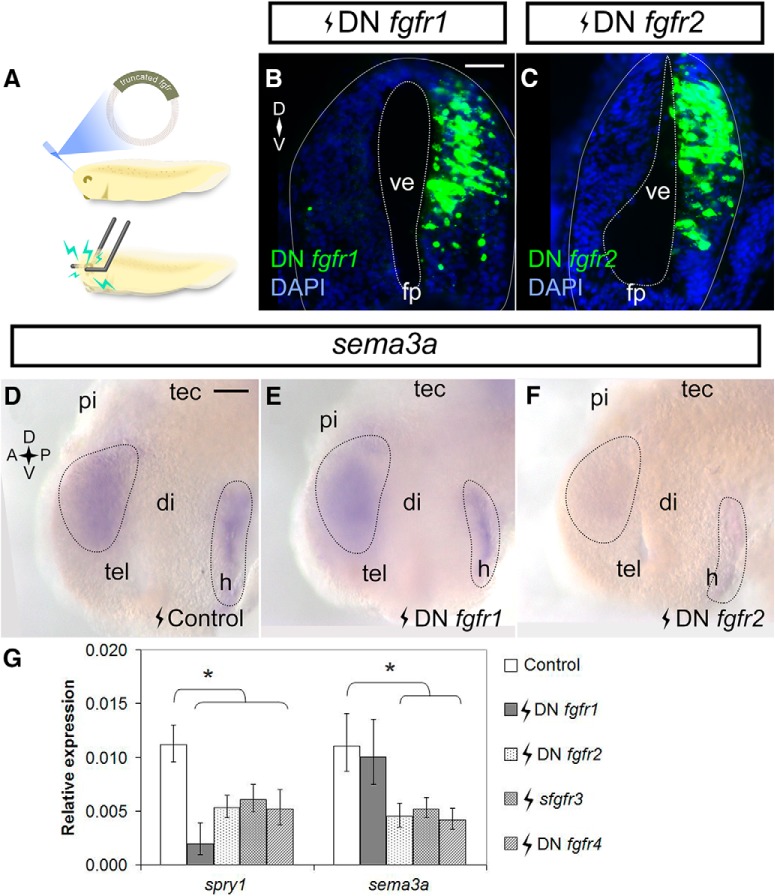
Fgfr2-4 inhibition downregulates *sema3a* in the forebrain. ***A***, A cartoon of stage 27/28 forebrain electroporation. ***B***, ***C***, Embryos were electroporated with *pCS2*-*DNfgfr1* (***B***) and *pCS108*-*DNfgfr2* (***C***) and processed in transverse cryostat sections by FISH for *fgfr1/2* at stage 32 to confirm transgene expression. ***D–F***, Embryos were electroporated with control *pCS2-GFP* (***D***, *n* = 37 brains, *N* = 3), *pCS2*-*DNfgfr1* (***E***, *n* = 20 brains, *N* = 3), or *pCS108*-*DNfgfr2* (***F***, *n* = 16/24 brains had decreased expression, *N* = 3) and processed for *sema3a* expression by wholemount ISH at stage 32. ***G***, *sema3a* mRNA levels in brains electroporated with truncated *fgfrs* was measured by RT-qPCR. *spry1* was a readout of Fgfr inhibition. Bars represent mean ± SEM for control (*n* = 32, *N* = 4), DN *fgfr1* (*n* = 24, *N* = 4), DN *fgfr2* (*n* = 20, *N* = 3), *sfgfr3* (*n* = 30, *N* = 4), and DN *fgfr4* (*n* = 31, *N* = 4); *p < 0.05 statistical significance versus the control was determined using the REST algorithm. Scale bars, 50 µm. di, diencephalon; fp, floor plate; h, hypothalamus; pi, pineal gland; tec, optic tectum; tel, telencephalon; ve, ventricle.

To determine whether similar Fgfr2/3-*sema3a* regulatory relationship occurs in the embryonic brain, expression vectors for DN *fgfr1/2/4* and *sfgfr3* were electroporated into stage 27/28 *X. laevis* forebrains (Fig. 3*A*). Twenty-four hours after electroporation, the forebrains were collected for ISH and RT-qPCR to spatially and quantitatively assess gene expression, respectively. The DN *fgfr1/2* constructs had strong expression in the electroporated half of the forebrain, as assayed by FISH (Fig. 3*B*,*C*), as did *sfgfr3* and DN *fgfr4* vectors (data not shown). We first assessed *sema3a* expression in electroporated brains by ISH (Fig. 3*D*–*F*). As in XTC cells, expression of DN *fgfr1* (*n* = 20 brains, *N* = 3) did not noticeably affect *sema3a* compared to the *pCS2-GFP* control (*n* = 37 brains, *N* = 3), but was shown previously to repress forebrain *slit1* expression (Yang et al., 2018). In contrast, by ISH, *sema3a* expression in DN *fgfr2* electroporated brains was decreased relative to control (*n* = 16/24 and *N* = 3 DN *fgfr2* brains). RT-qPCR verified that, compared to control electroporation (*n* = 121 brains, *N* = 4; Fig. 3*G*), DN *fgfr1* did not affect *sema3a* significantly (*n* = 24 brains, *N* = 4, *p* = 0.754; row g, Table 2), whereas DN *fgfr2*, *sfgfr3*, and DN *fgfr4* decreased *sema3a* induction to 40.6% (*n* = 20 brains, *N* = 3, *p* = 0.005; row h, Table 2), 47.1% (*n* = 30 brains, *N* = 4, *p* = 0.004; row i, Table 2), and 37.9% (*n* = 31 brains, *N* = 4, *p* < 0.001; row j, Table 2), respectively. Of note, all the truncated Fgfrs were effective at blocking Fgf signaling as seen by the decreased expression of *spry1* by RT-qPCR, a feedback regulator of Fgfr signaling (Hacohen et al., 1998; Atkinson-Leadbeater et al., 2009). In agreement with the XTC cell line data, we find that Fgfr2/3 regulate forebrain *sema3a*. While Fgfr4 regulates *sema3a* in the forebrain, *fgfr4* mRNA is absent in XTC cells. In contrast, Fgfr1 appears to be the sole Fgfr regulating *slit1* in the forebrain (Yang et al., 2018). Thus, *sema3a* and *slit1* mRNA levels are maintained by distinct Fgfrs in the embryonic *Xenopus* forebrain.

### Pharmacological blockade of PI3K signaling inhibits both *sema3a* and *slit1* expression

**Figure 4.**
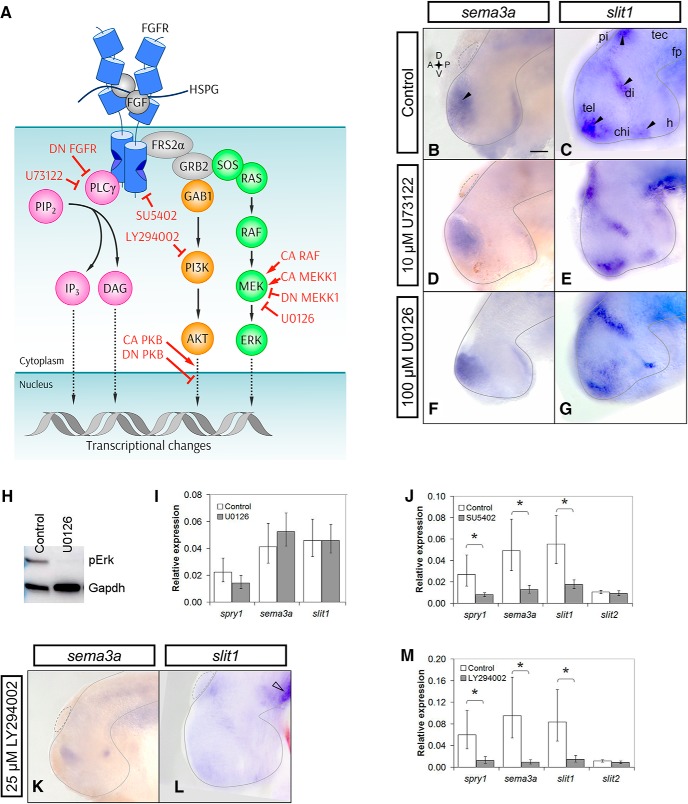
PI3K inhibition decreases both *sema3a* and *slit1* expression in the forebrain. ***A***, The canonical sequence of Fgf signaling intermediates is indicated by solid arrows. Dotted arrows show further signaling through downstream factors (Rodriguez-Viciana et al., 1994; Zimmermann and Moelling, 1999; Jiminez et al., 2002; Nakayama et al., 2008; Aksamitiene et al., 2012; Mendoza et al., 2011; Chen et al., 2012; Selvaraj et al., 2014). The activated complex of HSPG-FGF-FGFR dimerizes to phosphorylate the FGFR intracellular tails and recruits FRS2α, GRB2, GAB1, SOS, and RAS (Rubinfeld and Seger, 2004; Lemmon and Schlessinger, 2010). GAB1 activates the PI3K-AKT relay (orange). RAS activates the RAF-MEK-ERK cascade, i.e., MAPK signaling (green). PLCγ can directly bind to the phosphorylated FGFR to hydrolyze PIP_2_ to IP_3_ and DAG (magenta). The inhibitors against FGFRs, MEK, PI3K, and PLCγ are SU5402, U0126, LY294002, and U73122, respectively. CA RAF and CA MEKK1 were used to overactivate the ERK MAPK pathway. DN MEKK1 was used to inhibit the MAPK pathway. The CA and DN PKB (AKT) constructs were used for gain and loss of function of AKT signaling, respectively. Wild-type PLCγ was overexpressed to increase PLCγ signaling, and the PLCγ-deficient DN FGFR was used to inhibit PLCγ activation. ***B***–***G***, Stage 32 brains were treated with control (***B***, *n* = 46 brains, *N* = 4; ***C***, *n* = 39 brains, *N* = 3) or 10 µM U73122 solution and processed by wholemount ISH for *sema3a* (***D***, *n* = 12 brains, *N* = 3) and *slit1* (***E***, *n* = 11 brains, *N* = 2). Black arrowheads indicate the *sema3a* and *slit1* domains of interest. Mek inhibition with 100 µM U0126 treatment did not visibly decrease *sema3a* (***F***, *n* = 11 brains, *N* = 2) or *slit1* (***G***, *n* = 11 brains, *N* = 2) expression by wholemount ISH. ***H***, Phospho-Erk (pErk) knockdown in U0126-treated forebrains confirmed by Western blot analysis (*n* = 18 brains and *N* = 3 for both treated and control). ***I***, ***J***, RT-qPCR of *sema3a* and *slit1* forebrain mRNA following treatment with U0126 (***I***, *n* = 23 brains and *N* = 4 for both U0126 and control) and 100 µM SU5402 (***J***, *n* = 17 brains and *N* = 3 for both SU5402 and control). ***K***–***M***, PI3K inhibition with 25 µM LY294002 treatment decreased *sema3a* (***K***, *n* = 15/20 LY294002 brains had decreased expression vs the control in ***B***, *N* = 3) and *slit1* (***L***, *n* = 11/15 LY294002 brains had decreased expression vs the control in ***C***, *N* = 2) expression by ISH. *slit1* expression in the floor plate (***L***, unfilled arrowhead) was unaffected by LY294002 treatment. ***M***, RT-qPCR for *sema3a* and *slit1* mRNA with LY294002 treatment (*n* = 11 brains and *N* = 2 for both LY294002 and control). In all RT-qPCR data, bars represent the mean ± SEM; *p < 0.05 using the REST algorithm for statistical significance. Scale bar, 50 µm. chi, optic chiasm; di, diencephalon; fp, floor plate; h, hypothalamus; pi, pineal gland; tec, optic tectum; tel, telencephalon.

Because we find that Fgf signaling regulates *sema3a* and *slit1* transcription *via* different Fgfr isoforms, we next asked whether distinct downstream signaling cascades are involved. The candidate pathways downstream of FGFRs are the MAPK, PI3K-AKT, and PLCγ cascades (Alessi et al., 1996; Alessi and Cohen, 1998; Klint and Claesson-Welsh, 1999; Rubinfeld and Seger, 2004; Goetz and Mohammadi, 2013; Ornitz and Itoh, 2015; Nakamura and Fukami, 2017; Fig. 4*A*). To determine which of these signaling pathways are relevant for *sema3a* and *slit1* expression, pharmacological inhibitors against each pathway were applied to exposed brain preparations of stage 32 *X. laevis* embryos (Chien et al., 1993; Atkinson-Leadbeater et al., 2010). The pharmacological approach has the advantage that pathways can be inhibited for a short time period (hours), where effects on gene transcription are likely directly attributable to changes in the activity of the pathway. The skin and dura overlying the brain on the left side of the embryo were surgically removed and the embryo incubated for 5 h in a control solution or media containing the appropriate inhibitor. Notably, the Fgfr inhibitor SU5402 decreases *sema3a* and *slit1* expression over this same time period (Atkinson-Leadbeater et al., 2010). We used the Mek inhibitor U0126 (100 µM) to inhibit the Erk MAPK pathway, LY294002 (25 µM) to block PI3K, and U73122 (10 µM) to inhibit PLCγ (Fig. 4*A*). These concentrations were shown previously to be effective in *Xenopus* and zebrafish *in vivo* (Westfall et al., 2003; Vergara and Del Rio-Tsonis, 2009; Belgacem and Borodinsky, 2011; Stulberg et al., 2012; Das et al., 2016). In all cases, the control embryos were exposed to the same concentration of DMSO solvent used to dissolve the drugs. We assessed both the spatial patterns and the levels of *sema3a* and *slit1* expression following inhibitor treatment by wholemount ISH and RT-qPCR, respectively.

By ISH, embryos treated with the PLCγ inhibitor U73122 showed no obvious change when comparing the control treatment (*n* = 46 brains, *N* = 4;  Fig. 4*B*, *n* = 39 brains, *N* = 3; Fig. 4*C*) with that of U73122 (*n* = 12 brains, *N* = 3; Fig. 4*D*, *n* = 11 brains, *N* = 2; Fig. 4*E*). Similarly, inhibition of the MAPK pathway produced no obvious qualitative differences in the expression of *sema3a* (*n* = 11 brains, *N* = 2;  Fig. 4*F*) and *slit1* (*n* = 11 brains, *N* = 2; Fig. 4*G*). Importantly, U0126 effectively blocked Mek activity in this *in vivo* assay, as assessed by Western blot analysis of the phosphorylated form of Erk in control and inhibitor-treated brains (*n* = 18 brains and *N* = 3 for both treated and control; Fig. 4*H*). We used RT-qPCR to verify the lack of change in the *sema3a* and *slit1* ISH signal we observed. In agreement with the ISH data, no significant changes in *sema3a* (*p* = 0.61; row k, Table 2) and *slit1* (*p* = 0.99; row l, Table 2) levels were observed by RT-qPCR with U0126 treatment (*n* = 23 brains and *N* = 4 for both U0126 and control; Fig. 4*I*). As a positive control for RT-qPCR we confirmed that *sema3a* and *slit1* levels were decreased after 5 h of Fgfr inhibition by SU5402 (100 µM), as we had observed previously by ISH (Atkinson-Leadbeater et al., 2010): SU5402 blocked Fgfr signaling, shown by decreased *spry1* (29% of control; *n* = 17 brains and *N* = 3 for both SU5402 and control, *p* = 0.049; Fig. 4*J*; row m, Table 2), and lowered *sema3a* and *slit1* levels to 26% (*p* = 0.015; row n, Table 2) and 32% (*p* = 0.012; row o, Table 2) of that of control, respectively. Importantly, however, mRNA levels of *slit2*, whose expression in the dorsal diencephalon we found previously to be Fgf-independent (Atkinson-Leadbeater et al., 2010), were not significantly affected by SU5402 (*p* = 0.410; row p, Table 2).

With PI3K pathway blockade by LY294002, however, there was visible reduction of signal for both *sema3a* (*n* = 15/20 LY294002 brains, *N* = 3; Fig. 4*B*,*K*) and *slit1* (*n* = 11/15 LY294002 brains, *N* = 3; Fig. 4*C*,*L*). Levels of *sema3a* in the forebrain were visibly lowered, while *slit1* was reduced in the ventral telencephalon, hypothalamus, diencephalon, and adjacent to the pineal gland. Interestingly, ISH of *slit1* expression in the ventral neural tube caudal to the midbrain was not noticeably different in LY294002-treated and control brains, suggesting a different mechanism of *slit1* regulation in this brain region. When assessed quantitatively by RT-qPCR, LY294002 decreased *sema3a* and *slit1* to 9.5% (*p* = 0.002; row q, Table 2) and 17% (*p* = 0.022; row r, Table 2) of control levels, respectively, while *slit2* (*p* = 0.255; row s, Table 2) was unchanged (*n* = 11 brains and *N* = 2 for both LY294002 and control; Fig. 4*M*). Together, these data suggest that the PI3K pathway positively regulates *sema3a* and *slit1* expression in the forebrain. Interestingly, *spry1* was unaffected by U0126 (*p* = 0.454; row t, Table 2), but downregulated by SU5402 (*p* = 0.049; row u, Table 2) and LY294002 (*p* = 0.041; row v, Table 2), suggesting that Spry1 may be a feedback regulator that functions specifically downstream of PI3K. In agreement, LY294002 downregulates *Spry1* expression in bovine granulosa cells (Jiang et al., 2011; Jiang and Price, 2012). Therefore, the ISH and RT-qPCR data support the idea that Fgfrs promote *sema3a* and *slit1* transcription in the forebrain via PI3K signaling. The speed of the dysregulation with LY294002 argues against cell death or proliferation as explanations for the decreased guidance cue expression.

To support the idea that the PI3K pathway acts downstream of Fgfr signaling to maintain *sema3a* and *slit1* mRNA levels, we first assessed PI3K pathway activation in the developing forebrain with Fgfr inhibition. Brains were exposed at stage 33/34 to the Fgfr inhibitor SU5402 (100 µM) and were collected 5 h later, and processed by Western blot analysis for the phosphorylated form of Akt (pAkt), a readout of PI3K pathway activation. pAkt levels were downregulated with SU5402 as compared to control (data not shown), indicating that Fgfr forebrain signaling promotes PI3K pathway activity.

### Molecular blockade of PI3K signaling inhibits both *sema3a* and *slit1* expression

**Figure 5.**
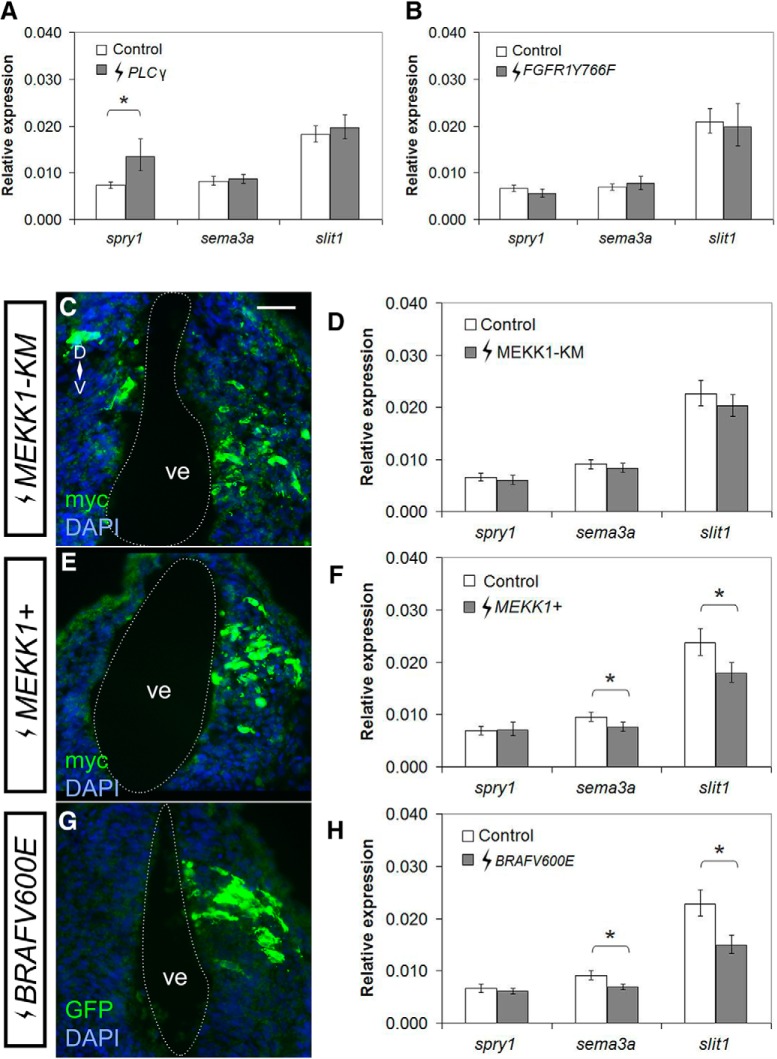
Activation of the MAPK pathway is sufficient to inhibit both *sema3a* and *slit1* expression in the forebrain. Constructs for Fgfr downstream signaling intermediates were electroporated into the forebrain of stage 27/28 embryos, and *spry1*, *sema3a*, and *slit1* expression in the forebrain were quantified by RT-qPCR 24 h after electroporation (stage 32). ***A***, ***B***, RT-qPCR when PLCγ signaling was augmented by *pEYFP-PLCγ* (***A***, *PLCγ n* = 32 brains, *N* = 4 vs control *n* = 31, *N* = 4), and inhibited with the PLCγ-deficient FGFR1, *phRluc-N1-FGFR1Y766F* (***B***, *FGFR1Y766F n* = 22 brains, *N* = 3 vs control *n* = 24, *N* = 3). ***C***–***G***, The MAPK pathway was inhibited with a DN *MEKK1*, *pCS2-MEKK1-KM*, and activated with either *pCS2-MEKK1+* or *pCS108-BRAFV600E*. Immunohistochemistry on transverse sections of the forebrain against myc (***C***, ***E***) and GFP (***G***) tags confirm successful electroporations. Changes in gene expression was quantified by RT-qPCR after electroporation with *MEKK1-KM* (***D***, *n* = 30, *N* = 4), *MEKK1+* (***F***, *n* = 28, *N* = 4), and BRAFV600E (***H***, *n* = 32, *N* = 4) and compared to control *pCS2-GFP* electroporation (***D***, ***F***, ***H***, *n* = 31, *N* = 4). Bars represent the mean ± SEM; *p < 0.05 using the REST algorithm for statistical significance. Scale bar, 50 µm. ve, ventricle.

To confirm the pharmacological inhibition data, we turned to a molecular approach to disrupt the candidate pathways through electroporation of plasmids into the stage 27/28 brain that allowed for the gain and loss of function of specific signaling intermediates (Fig. 4*A*). Forebrain *sema3a* and *slit1* mRNA levels were assessed by RT-qPCR 24 h after electroporation. In agreement with the U73122 data, molecular manipulation of the PLCγ pathway did not affect *sema3a* and *slit1* expression. A plasmid expressing PLCγ did not affect *sema3a* (*p* = 0.520; row w, Table 2) and *slit1* (*p* = 0.594; row x, Table 2) levels; however, PLCγ overexpression increased *spry1* levels to almost twice (181%, *p* = 0.016; row y, Table 2) that of control, suggesting strong Fgf signal activation (*PLCγ n* = 32 brains and *N* = 4 vs control *n* = 31 brains and *N* = 4; Fig. 5*A*). Furthermore, preventing PLCγ activation downstream of Fgfr1, by expression of the *phRluc-N1-FGFR1Y766F* construct that encodes for human FGFR1 mutated at Tyr766 to prevent binding to PLCγ (Browaeys-Poly et al., 2010), failed to change *sema3a* (*p* = 0.351; row z, Table 2) and *slit1* (*p* = 0.654; row aa, Table 2) levels (*FGFR1Y766F n* = 22 brains and *N* = 3 vs control *n* = 24 brains and *N* = 3; Fig. 5*B*).

Similarly, in agreement with the MAPK U0126 data, molecular blockade of the pathway by electroporation with DN *MEKK1* (Fig. 5*C*), *pCS2-MEKK1-KM* (Ben Messaoud et al., 2015), produced no change in *sema3a* (*p* = 0.478; row ab, Table 2) or *slit1* (*p* = 0.372; row ac, Table 2) expression (*MEKK1-KM n* = 30 brains and *N* = 4 vs control *n* = 31 brains and *N* = 4; Fig. 5*D*). Interestingly, the MAPK cascade appears to be sufficient to control *sema3a* and *slit1* transcription. Electroporation of constitutively active (CA) *MEKK1* (Fig. 5*E*), *pCS2-MEKK1+* (Ben Messaoud et al., 2015), downregulated *sema3a* and *slit1* mRNA to 80.7% (*p* = 0.023; row ad, Table 2) and 76.1% (*p* = 0.041; row ae, Table 2) of control electroporation (*MEKK1+ n* = 28 brains and *N* = 4 vs control *n* = 31 brains and *N* = 4; Fig. 5*F*). Likewise, CA *B-RAF* (*pCS108-BRAFV600E*; Boehm et al., 2007; Tachibana et al., 2016) electroporation (Fig. 5*G*) decreased *sema3a* and *slit1* expression to 76.2% (*p* = 0.008; row af, Table 2) and 65.9% (*p* < 0.001; row ag, Table 2) of control electroporation, respectively (*BRAFV600E n* = 32 brains and *N* = 4 vs control *n* = 31 brains and *N* = 4; Fig. 5*H*). Of note, these changes in gene expression were likely underestimated in that only half of the brain generally expressed the transgenes after electroporation. Thus, MAPK activation is sufficient but not necessary to control *sema3a* and *slit1*, and in a manner distinctive of Fgfr-PI3K signaling, as *spry1* expression was unaffected. In summary, the molecular data confirm that neither the MAPK nor PLCγ pathways are required to maintain *sema3a* and *slit1* expression.

**Figure 6.**
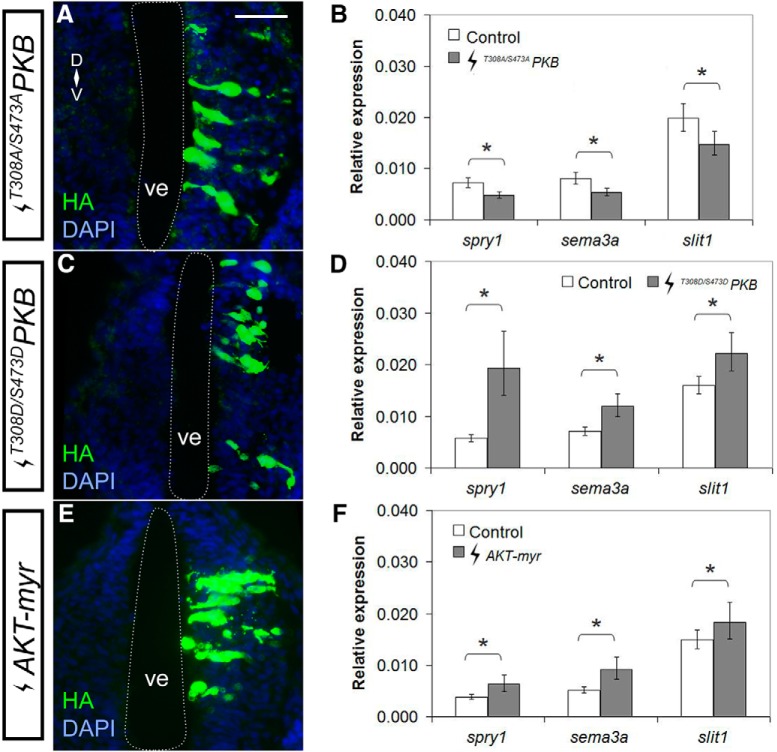
Akt signaling positively regulates *sema3a* and *slit1* in the forebrain. Plasmids encoding DN *AKT* (***A***, ***B***) *^T308A/S473A^PKB*, and CA *AKT* (***C***, ***D***) *^T308D/S473D^PKB*, and (***E***, ***F***) *AKT-myr* were electroporated into the forebrain of stage 27/28 embryos. ***A***, ***C***, ***E***, Forebrain transgene expression was assessed by immunohistochemistry for HA-tagged AKT mutants. ***B***, ***D***, ***F***, *spry1*, *sema3a*, and *slit1* expression in the forebrain 24 h after electroporation was quantified by RT-qPCR for (***B***) *^T308A/S473A^PKB* (*n* = 21, *N* = 3 vs control *n* = 23, *N* = 3), (***D***) *^T308D/S473D^PKB* (*n* = 40, *N* = 5 vs control *n* = 39, *N* = 5), and (***F***) *AKT-myr* (*n* = 22, *N* = 3 vs control *n* = 24, *N* = 3), where the control electroporation was *pCS2-GFP*. Bars represent the mean ± SEM; *p < 0.05 using the REST algorithm for statistical significance. Scale bar, 50 µm. ve, ventricle.

In contrast, molecular modulation of PI3K-Akt signaling affected expression of these two guidance cues. By RT-qPCR, DN *AKT* electroporation (Fig. 6*A*), *^T308A/S473A^PKB* (Palmada et al., 2005) reduced *sema3a* and *slit1* to 66.7% (*p* = 0.022; row ah, Table 2) and 74.4% (*p* = 0.007; row ai, Table 2) of control levels, respectively (*^T308A/S473A^PKB n* = 21 brains and *N* = 3 vs control *n* = 23 brains and *N* = 3; Fig. 6*B*). The RT-qPCR data for Akt loss of function is consistent with the ISH and RT-qPCR data showing that PI3K inhibition reduces *sema3a* and *slit1* levels. In contrast, CA *AKT* electroporation (Fig. 6*C*), *^T308D/S473D^PKB*, AKT with activating phosphomimetic mutations (Palmada et al., 2005), upregulated *sema3a* and *slit1* in the forebrain to 158% (*p* = 0.001; row aj, Table 2) and 131% (*p* = 0.043; row ak, Table 2) of control, respectively (*^T308D/S473D^PKB n* = 40 brains and *N* = 5 vs control *n* = 39 brains and *N* = 5; Fig. 6*D*). Similarly, *AKT-myr* electroporation (Fig. 6*E*), a myristoylated CA AKT (Jin et al., 2015), upregulated *sema3a* and *slit1* to 175% (*p* = 0.001; row al, Table 2) and 123% (*p* = 0.019; row am, Table 2) of control, respectively (*n* = 22 brains and *N* = 3 vs control *n* = 24 brains and *N* = 3; Fig. 6*F*). Importantly, modulation of Akt activity resulted in corresponding changes in the levels of the Fgfr pathway feedback regulator, *spry1*, whereby CA *AKT* increased transcription (318%, *p* = 0.004; row an, Table 2) and DN *AKT* repressed transcription (67.8%, *p* = 0.041; row ao, Table 2). The fact that changes in this known Fgfr downstream effector are observed with PI3K-Akt manipulation both molecularly and pharmacologically, argues that Fgfr signaling regulates *sema3a* and *slit1* through the PI3K-Akt pathway.

### Inhibition of PI3K signaling *in vivo* disrupts RGC axon guidance at the mid-diencephalic turn

Previously, we found that downregulation of *sema3a* and *slit1* expression, directly through brain electroporation of antisense oligonucleotides or indirectly via pharmacological inhibition of Fgf signaling with SU5402, caused RGC axons to stall in the mid-diencephalon and fail to move on toward their dorsal midbrain target, the optic tectum (Atkinson-Leadbeater et al., 2010). If Fgf signaling works through the PI3K pathway to help maintain *sema3a* and *slit1* guidance cues we expected a similar axon “stall” phenotype with PI3K pathway inhibition by application of LY94002 (20–25 µM) *in vivo* in the developing forebrain neuroepithelium. We performed the exposed brain preparation at stage 33/34, when the first RGC axons from the right eye enter the contralateral ventral diencephalon before extending dorsally through the diencephalon. Axon guidance was assessed by anterogradely labeling RGC axons by HRP at stage 40, when normally the vast majority of RGC axons have grown into and innervated the optic tectum (Holt, 1989).

**Figure 7.**
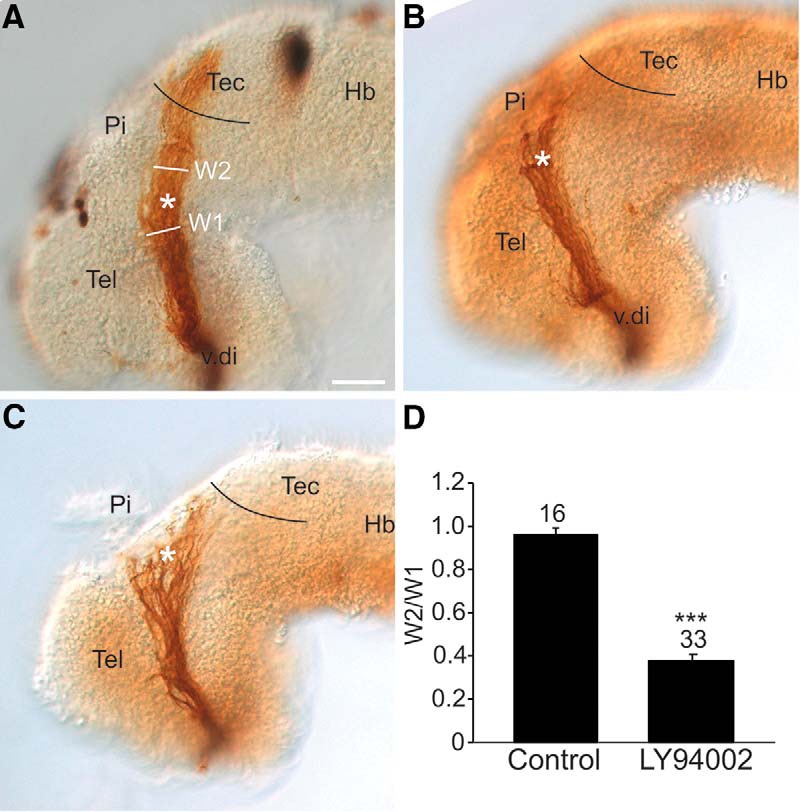
PI3K signaling regulates the behavior of RGC axons at the mid-diencephalic turn. Lateral views of stage 40 wholemount brains with HRP-labeled optic tracts that were exposed at stage 33/34 either to (***A***) control DMSO solution (*n* = 16 brains, *N* = 3) or (***B***, ***C***) 20 µM LY294002 (*n* = 33 brains, *N* = 3). Black line represents the approximate anterior border of the optic tectum, and the asterisk the location of the mid-diencephalic turn guidance choice point. ***D***, The RGC axon stall phenotype was quantified by representing the width of the optic tract post-mid-diencephalic turn (W2) as a ratio (W2/W1) to the width of the optic tract (W1) at the mid-diencephalic turn. On each bar is the number of individual brains pooled from 3 independent experiments. Bars represent the mean ± SEM; ***p < 0.001 using the unpaired Student’s *t* test. Scale bar, 50 µm. Hb, hindbrain; Pi, pineal gland; Tec, tectum; Tel, telencephalon; v.di, ventral diencephalon.

As expected, in control DMSO brains RGC axons targeted the optic tectum (*n* = 16 brains, *N* = 3; Fig. 7*A*). In contrast, when brains were exposed to LY94002 6 h before the first RGC axons reach the mid-diencephalon, many of the optic tracts had significant numbers of axons that stalled in the mid-diencephalon and failed to navigate to the optic tectum, similar to what we observed previously with Fgf signaling inhibition (*n* = 33 brains, *N* = 3; Fig. 7*B*,*C*; Atkinson-Leadbeater et al., 2010). Of note, given the likelihood that the PI3K pathway has additional roles in the formation of the optic projection, the LY294002 phenotype was not identical to that produced with the Fgfr inhibitor; for instance, the LY294002 optic tract appeared defasciculated (Fig. 7*C*), a phenotype not observed with SU5402.

We quantified the stall phenotype by measuring the width of the optic tract at the mid-diencephalic turn, and then the widest portion of the optic tract distal to the turn, and represented these widths as a ratio. While in control embryos the tract was generally the same width at the two locations, in PI3K inhibitor-treated brains, the optic tract was significantly (*p* < 0.001; row ap, Table 2) narrower post-turn, reflecting the fall-out of axons at the mid-diencephalon (Fig. 7*D*). These data support the idea that PI3K works downstream of Fgfrs to help maintain the expression of Sema3a and Slit1, two repellents key for controlling the trajectories of RGC axons at the mid-diencephalic choice point. We propose that guidance of RGC axons at the mid-diencephalon depends on PI3K signaling in neuroepithelial cells maintaining Sema3a/Slit1 levels, and not PI3K signaling in the RGC growth cones themselves. In support, similar to what was observed with LY294002 brain exposures, inhibition of PI3K signaling only in brain neuroepithelial cells and not RGC axons, via electroporation of DN *AKT* into stage 28 brains, produced an HRP-labeled optic projection that unlike GFP controls was significantly narrower after the mid-diencephalic turn (*p* < 0.0001; row aq, Table 2, *^T308A/S473A^PKB n* = 16 brains and *N* = 3 vs control *n* = 16 brains and *N* = 3). Of note, a prior pharmacological screen we performed failed to identify a role for the PI3K pathway in guiding RGC axons within the diencephalon (Webber et al., 2005). We feel this discrepancy reflects the nature of the simplified read-outs in the initial screen of mis-routing of axons from the optic tract and failure of optic tectum innervation. The “axon stalling” in the mid-diencephalon we characterized for the first time with Fgfr inhibition (Atkinson-Leadbeater et al., 2010), and observed here with PI3K inhibition, is often partial in nature, where some axons escape and go on to innervate the optic tectum. At the time of the screen, we would have classified these “partial stall” optic tracts as normal with regards to “guidance,” because axons did reach the optic tectum and misrouting of axons from the optic tract was not observed. Thus, the PI3K inhibitor data provide additional support that Fgf signaling works through the PI3K pathway to maintain expression of guidance cues important for guidance of RGC axons in the diencephalon.

## Discussion

Guidance cues direct the trajectory of growing axons so that proper synaptic connections can be made. While families of guidance cues have been identified, their transcriptional regulation has been largely unexplored nor has the co-regulation of two distinct guidance cues at the same axon choice point been considered. Previously, we found that guidance of the axons of the optic tract relies on repellents Sema3a and Slit1 expressed in the forebrain (Atkinson-Leadbeater et al., 2010). Building on our prior work (Yang et al., 2018), here we find that *sema3a* and *slit1* genes are expressed in essentially non-overlapping domains of the forebrain, and are regulated by distinct Fgfrs. Despite these differences, our data support the idea that transactivation of *sema3a* and *slit1* converges along the PI3K-Akt pathway. Thus, we identify for the first time *in vivo* the extracellular receptors and signal transduction pathway that regulate two key guidance cues at a single brain decision point, and suggest a common intrinsic mechanism.

Although *sema3a* and *slit1* both depend on Fgf signaling for expression, and together repel RGC axons at the same mid-diencephalic guidance choice point (Atkinson-Leadbeater et al., 2010), our data argue that distinct Fgfrs control the expression of the two genes. Fgfr2-4 are key regulators of *sema3a* transcription, while Fgfr1 promotes *slit1* expression (Yang et al., 2018). One possibility is that the choice of receptors simply reflects the differential expression of *fgfrs* in separate cell populations. *slit1* mRNA is largely present in cells at the pial surface of the brain, which are likely post-mitotic, though it does overlap significantly with *fgfr1* in ventricular progenitors in the anterior forebrain, and in the floor plate more caudally (Yang et al., 2018). In contrast, *sema3a* mRNA is present within the progenitor ventricular zone. Thus, the distinct *sema3a*/*slit1* cell populations may express different Fgfr isoforms to respond to the specific Fgfs available locally. Two pieces of data argue against this possibility. First, *fgfr1* overlaps extensively with both *sema3a* and *slit1*, yet specific inhibition of Fgfr1 only reduces *slit1* mRNA levels. Second, the forebrain data are consistent with those from cell culture where *slit1* is regulated by Fgfr1 (Yang et al., 2018) and *sema3a* by Fgfr2/3. These data argue that distinct Fgfrs control the expression of specific guidance genes. The retinal expression data support this idea, in that only *fgfr1* shows extensive overlap with that of *slit1* in differentiating RGCs, whereas mRNA for all four receptors is present in the *sema3a*-expressing cells of the lens (data not shown). Thus, while multiple Fgfrs appear to work together to promote *sema3a* expression, only Fgfr1 regulates *slit1* expression in the forebrain and in nonneuronal cell cultures (Yang et al., 2018).

Interestingly, our experiments suggest that despite the differential control of *sema3a* and *slit1* by Fgfrs, downstream regulation converges along the same PI3K-Akt pathway. Similar to inhibition of Fgfr signaling, we find that multiple means of inhibiting the PI3K pathway, including pharmacological inhibition and molecular Akt gain and loss of function, reduce significantly *sema3a* and *slit1* forebrain expression. We propose that Fgf and PI3K signaling regulate *sema3a* and *slit1* expression cell-autonomously, based on gene expression, and the speed (hours) of the changes in gene expression with pharmacological inhibition of Fgfrs (Atkinson-Leadbeater et al., 2010) and PI3K *in vivo*. While we cannot exclude the possibility that Fgfrs and PI3K work in parallel pathways, our data are supportive of the idea that the PI3K pathway works downstream of Fgfrs. First, pAkt analysis indicates that the PI3K pathway is regulated positively by Fgfr signaling in the forebrain. In agreement, we find that in the forebrain PI3K-Akt signaling acts on the Fgfr downstream target *spry1.* Second, other classical Fgfr signal transduction mechanisms do not appear to directly promote forebrain *sema3a* and *slit1* expression, pointing to the PI3K pathway as the Fgfr signaling mediator. The PI3K pathway is known to work downstream of many extrinsic signaling molecules (Manning and Cantley, 2007; Hemmings and Restuccia, 2012), and so it is also likely that while the pathway works with Fgfrs to help maintain *sema3a* and *slit1* expression, PI3K has other roles in the growth of axons in the forebrain, including regulating the expression of other key ligands in the brain neuroepithelium, and mediating signaling directly within axonal growth cones. In support, while the RGC axon phenotype observed with PI3K inhibition resembles that observed with Fgfr inhibition (Atkinson-Leadbeater et al., 2010), there are additional features, such as axon defasciculation.

The idea that multiple Fgfrs might work through the same PI3K pathway intermediates stands out from the current understanding of Fgfr downstream pathways. Although FGFR1-4 have similar structures, they can activate distinct sets of downstream factors and cell behaviors (Dailey et al., 2005; Ornitz and Itoh, 2015). For instance, transfection of Fgfr1 but not Fgfr4 leads to cell survival and growth of BaF3 murine lymphoid cells (Wang et al., 1994). Here, Fgfr1 strongly phosphorylates Erk, whereas Fgfr4 does not affect Erk and only weakly activates PLCγ. When *Fgfrs* are transfected into multiple mammalian cell lines, Fgfr1/3 strongly and consistently activate Erk1/2 and PLCγ, while Fgfr4 only weakly phosphorylates these effectors (Vainikka et al., 1994; Wang et al., 1994; Klint et al., 1995; Shaoul et al., 1995; Kanai et al., 1997; Raffioni et al., 1999). Instead, in cell lines the PI3K pathway may be the major pathway downstream of Fgfr4, as is the case for mouse NIH 3T3 fibroblasts (Vainikka et al., 1994). Thus, in contrast to the idea that differential Fgfr usage stems from a requirement for divergent downstream pathways, our data reveal that the distinct use of Fgfrs in the *Xenopus* forebrain to regulate different guidance genes converges on a single signal transduction pathway, PI3K-Akt. What remains to be elucidated is whether Fgfr2-4 display distinct amplitudes and durations of PI3K-Akt activation and thus distinct potencies in promoting *sema3a* transcription.

A question that arises is why Fgfr1 does not regulate *sema3a* in forebrain and XTC cells, given that *fgfr1* mRNA is present in both cell populations? Possibly, distinct Fgfrs access separate pools of a common downstream signaling factor. A plausible scenario is that scaffold proteins preserve the identity of the specific Fgfr from which an Fgf signal originates, even when Fgfrs share the same downstream signaling factors (Meister et al., 2013; Casar and Crespo, 2016). The Fgfr1-generated pool of PI3K may be only available to promote *slit1* and not *sema3a* transcription. These data provide support for the idea that a signaling molecule such as PI3K may be partitioned within a cell based on the receptor system for which they act downstream. While multiple MAPK scaffold proteins have been identified (Casar and Crespo, 2016), we are aware of only one scaffold protein identified for PI3K-AKT, IQGAP1, which also scaffolds MAPK components under the control of epidermal growth factor receptor (Roy et al., 2005; Chen et al., 2010; Choi and Anderson, 2016, 2017).

Some studies have considered the intracellular regulation of guidance genes in cultured cell lines and after injury, but endogenous control of guidance gene expression *in vivo* in embryonic neuroepithelial cells has not been addressed. The *in vitro* studies implicate predominantly the MAPK pathway, though the time frames (days) examined are much longer than the 5-h time window we investigated. For instance, depletion of dual leucine zipper kinase, a MAPKKK that preferentially activates Jnk but may also activate Erk and p38 MAPKs, increases the expression of guidance receptors *Epha7*, *Nrp1*, *Plxna4*, and *Unc5a*, and downregulates the expression of the ligand *Sema6b* in cultured mouse Neuro-2a neuroblastoma cells (Blondeau et al., 2016). Further, in an ischemic retinopathy model, Interleukin 1 Beta activation of proteinase-activated receptor-2 works through Erk/Jnk in mouse RGCs to decrease *Sema3a* expression (Sitaras et al., 2015). Inhibition of the MAPK pathway (short or long term), however, has no effect on *Xenopus* embryonic forebrain *sema3a* and *slit1* levels. Yet, activation of the MAPK pathway is sufficient to repress *sema3a* and *slit1* transcription. These data argue that the MAPK pathway may only fine tune forebrain guidance cue levels rather than maintain expression. Since Fgf signaling increases *sema3a* and *slit1* expression (Atkinson-Leadbeater et al., 2010), it seems unlikely that MAPK silences *sema3a* and *slit1* downstream of Fgfrs but more likely downstream of other extrinsic molecule(s).

Guidance molecules have broad functions in development within and outside of the nervous system, and also in cancer (Chédotal et al., 2005; O’Donnell et al., 2009; Law and Lee, 2012; Luukko and Kettunen, 2016). This is true of the family of secreted Sema3s which similar to what we find for *Xenopus* RGCs (Atkinson-Leadbeater et al., 2010; Kita et al., 2013), function to repel and attract axons of distinct neuron types in various species (Fujisawa, 2004; Roth et al., 2009), as well as to promote axonal branching (Campbell et al., 2001) and to stimulate dendrite growth (Fenstermaker et al., 2004). Sema3s also mediate cardiovascular and bone development, lung branching, podocyte differentiation, and endothelial cell behavior (Feiner et al., 2001; Kagoshima and Ito, 2001; Yazdani and Terman, 2006; Reidy and Tufro, 2011) and can be either up- or downregulated in cancers (Chédotal et al., 2005). Thus, understanding how guidance cues like Sema3s are regulated is important given the diverse roles of these proteins in the developing and adult organism.

Axons do not rely on a single axon guidance cue to make turning decisions at guidance choice points. Instead, the actions of a large number of guidance ligands work cooperatively to ensure the proper behavior of axons. Our data indicate that at least for two guidance cues that work cooperatively to push RGC axons out of a mid-diencephalic intermediate target and on toward their final target, similar signaling mechanisms have evolved to control their expression. In brain neuroepithelial cells, the activation of Fgfrs and PI3K maintains Slit1 and Sema3a repellents in the guidance map within the mid-diencephalon. By having a common signaling mechanism, presumably the coordinated expression and function of such cues is facilitated. Multiple extrinsic factors likely define the precise spatiotemporal expression domains of *sema3a*, *slit1*, and other ligands important for guiding axons within the forebrain. Indeed, the fact that *slit2* mRNA within the diencephalon is not regulated by either PI3K or Fgfrs points to additional extrinsic factors and signaling mechanisms that control guidance cue expression, distinct from those regulating *sema3a* and *slit1.*

## Synthesis

Reviewing Editor: Robert Kalb, Northwestern University Decisions are customarily a result of the Reviewing Editor and the peer reviewers coming together and discussing their recommendations until a consensus is reached. When revisions are invited, a fact-based synthesis statement explaining their decision and outlining what is needed to prepare a revision will be listed below.Note: If this manuscript was transferred from JNeurosci and a decision was made to accept the manuscript without peer review, a brief statement to this effect will instead be what is listed below.

Both reviewers found merit with this work although there were disparate views about its overall impact. Reviewer #2 felt the advance was small and he/she was particularly uncomfortable with the putative connection between enhanced FGFRs on Sema3a and the PI3K/AKT pathway. This is a difficult issue to tease apart especially in the in vivo preparation. The ms. has been re-written to soften the claims and this seems reasonable to me.
